# Two Adults with Adrenal Myelolipoma and 21-Hydroxylase Deficiency

**DOI:** 10.1155/2009/916891

**Published:** 2009-08-13

**Authors:** Ingrid Nermoen, Ivar Følling, Kjetil Vegge, Arne Larmo, Bjørn Gunnar Nedrebø, Eystein Sverre Husebye, Kristian Løvås

**Affiliations:** ^1^Department of Endocrinology, Akershus University Hospital, 1478 Lørenskog, Norway; ^2^Institute of Medicine, Faculty Division Akershus University Hospital, University of Oslo, 1478 Lørenskog, Norway; ^3^Department of Radiology, Akershus University Hospital, 1478 Lørenskog, Norway; ^4^Department of Medicine, Haukeland University Hospital, 5021 Bergen, Norway; ^5^Institute of Medicine, University of Bergen, 5021 Bergen, Norway

## Abstract

We present incidentally discovered adrenal myelolipomas in two adult males with untreated congenital adrenal hyperplasia (CAH). The patients had simple virilizing form of CAH due to mutations in the *CYP21* gene coding for 21-hydroxylase; one was heterozygous for the I172N mutation and the other compound heterozygous for the I172N and I2splice mutations. The masses were not removed since myelolipomas are considered benign tumors, and the tumor size did not increase during four- and nine-year observation periods. An adrenal myelolipoma is an important exception to the rule that large tumours should be removed. Untreated CAH with prolonged excessive ACTH stimulation might contribute to the growth of adrenal masses. CAH should be considered as a differential diagnosis of patients with adrenal masses or adrenal myelolipomas.

## 1. Introduction

Adrenal incidentalomas are common; the challenge is to rule out malignancy or excess hormone secretion [1]. Myelolipomas are benign endocrine inactive tumors composed of adrenal, adipose, and myeloid tissue, representing 1.5%–9% of adrenal incidentalomas; the radiological characteristic is round, sharp-demarcated lesion with negative attenuation values because of its high fat content [2–5]. Large adrenal masses should usually be removed, but myelolipomas usually do not need resection. However, many of the reported cases were diagnosed after adrenalectomy. Rarely, myelolipomas have been associated with hormone excess syndromes, such as congenital adrenal hyperplasia (CAH) [6–10]. We here report two cases, which illustrate that large adrenal myelolipomas need not to be surgically removed and that CAH must be considered in patients with adrenal incidentalomas. 

## 2. Case Report 1

In 1999 a 50-year-old man presented with a right adrenal mass 5.7 × 5.1 × 1.9 cm. Medical history revealed precocius puberty at age 4, and he was diagnosed with “Morbus Glandulae Suprarenalis” when he was 9. Urinary 17-ketosteroids were found elevated, but suppressible with dexamethasone. He was treated with cortisone acetate 12.5 mg thrice daily from age 9, but at 18 he stopped the medication, after which he felt that his general wellbeing is improving. After this time he was lost to follow-up. At the age of 37 he underwent radioiodine treatment for thyrotoxicosis. He was otherwise healthy, working full time, and was the father of one child.

**Figure 1:**
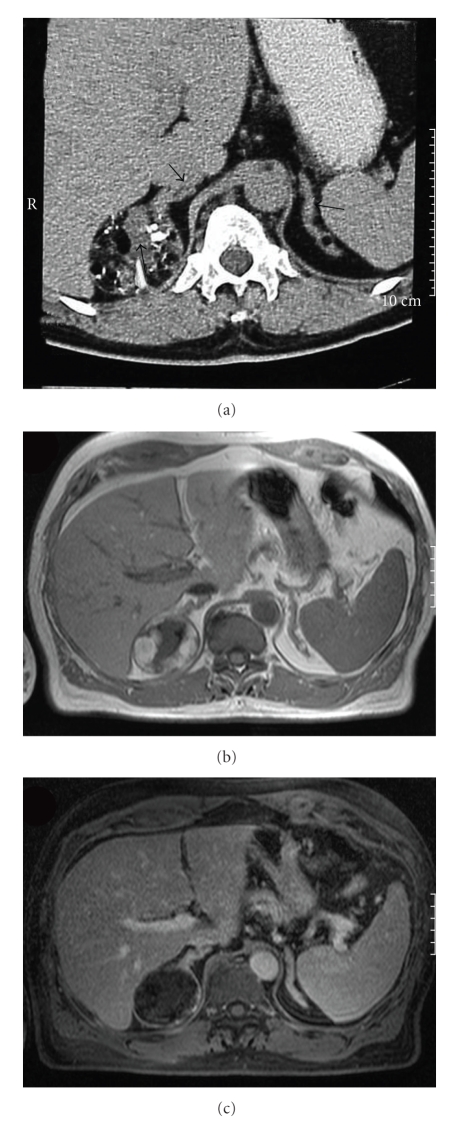
Computed tomography of the adrenals in Patient 1. (a) Arrows show a 5.7 centimetre large, expansive process in the right adrenal with a heterogeneous stroma, fat, calcifications, and in addition, pronounced bilateral adrenal hyperplasia. Magnetic resonance images of the adrenal tumor, T1 Weighted Imaging, with hyperintense fat (b) showing signal loss in fat suppressed image (c).

In spite of radioiodine treatment he felt he had persistent goitre, and his general practitioner referred him to a cervical CT, which due to his short stature included the upper abdomen. Pronounced bilateral adrenal hyperplasia and a 5.7 cm in diameter tumor described as a typical adrenal myelolipoma (capsule, heterogeneous stroma, and fat in the adrenal mass) was found (Figure 1). 

Further investigation revealed height 164 cm, weight 76 kg, and blood pressure 120/80 mmHg. On palpation the patient had no enlargement of the thyroid gland. Genital examination showed normal penile size and subnormal testicular volume of 12 mL bilaterally. Biochemical investigation revealed low basal cortisol, which did not increase after ACTH stimulation (Table 1). He had a high basal level of 17-OH-progesterone, typical of 21-hydroxylase deficiency, which was suppressed after a conventional dexamethasone test (Table 1). DNA sequencing of the *CYP21* (steroid 21-hydroxylase) gene revealed the c. 515T > A point mutation, which results in the predicted amino acid change I172N. The mutation was present in hemi- or homozygous form (i.e., the genotype was I 172N/deletion or I172N/I172N). The adrenal mass caused no symptoms and was considered highly likely benign and therefore left untreated. The tumor and hyperplasia did not change radiologically over a nine-year subsequent observation period. The patient continues to do well without steroid medication, but is instructed to take steroids during stress.

## 3. Case Report 2

A previously healthy 68-year-old man, father of one child, presented in 1994 with fatigue and nausea. For many years he had suffered arthralgic pain and had been treated with antiflogistics. The last months he had experienced problems with nocturia and impotence. He was referred to ultrasonography of the abdomen, on which a tumor in the right adrenal was detected. An adrenal CT scan showed a 4 × 4 × 2.5 cm tumor in the right adrenal with high fat content, which was radiologically considered to be a myelolipoma. The left adrenal was evaluated as normal. 

Further clinical investigation showed height 165 cm, blood pressure 140/80 mmHg, and normal male genitalia with testicular tenderness bilaterally. Biochemical investigation showed high basal level of 17-OH-progesterone, which was suppressible with dexamethasone, and an impaired cortisol response to ACTH stimulation (Table 1). Adrenal iodomethyl-19-norcholesterol scintigraphy showed high uptake in the right adrenal, which however was suppressed by dexamethasone; uptake was normal or low in the left adrenal. DNA sequencing of *CYP21* revealed compound heterozygozity for the I172N mutation (c. 515T > A) and the I2 splice mutation (g.655A/C > G, I172N/I2splice). 

The myelolipoma was not removed and the tumor size did not increase during a four-year observation period. The patient was started on treatment with 5 mg prednisolone daily for one year, later cortisone acetate 12.5 mg twice a day whereupon his condition improved. The patient died suddenly, 73 years old, in his home; the cause of death remains unknown.

## 4. Discussion

We here report two male patients who presented late in life with large adrenal myelolipomas. One was previously treated for CAH, whereas one was diagnosed with 21-hydroxylase deficiency after being diagnosed with the adrenal tumor at 68 years of age. Both tumors were left untreated and did not grow over five- and nine-year observation periods. 

An adrenal mass normally requires surgical removal if the largest diameter exceeds 3 cm [1]. Undiagnosed CAH must always be considered as a differential diagnosis to tumor, particularly with bilateral adrenal masses. Adrenal myelolipomas are benign and usually asymptomatic tumors that usually need not to be resected [2, 6, 7, 11]. Many of the cases that have been reported in literature were diagnosed after adrenalectomy [2, 6, 7, 11]. The imaging characteristics of myelolipomas usually allow presumptive diagnosis, but which may be confirmed by percutaneous needle biopsy in cases of extra-adrenal myelolipomas [5]. 

However, large myelolipomas could be dangerous if they start bleeding and give symptoms with pain, nausea, vomiting, and hypotension [2]. Because of mass effects and the risk of haemorrhage, elective removal of myelolipomas that exceed 10 cm in diameter might be considered. 

Increased prevalence of adrenal tumors has been reported in CAH patients and in heterozygote mutation carriers, possibly due to increased levels of ACTH which acts as a growth factor [12–15]. It has been suggested that inadequate glucocorticoid coverage in early life or frequent episodes of infection, both resulting in chronic ACTH increase, contribute to the development of adrenal tumors later in life [15, 16]. The aetiology is unknown, but several theories have been proposed, such as development from residual embryonic mesenchymal tissue in the adrenal glands, or metaplasia of reticuloendothelial cells as a result of chronic stress [2].

It was postulated early in the 1950s that ACTH elevation might induce transformation of adrenal tissue into myeloid tissue [17], and myelolipomas could thus be particularly associated with CAH. Hagiwara et al. investigated ACTH receptors and androgen receptor in a resected giant myelolipooma in a woman with CAH. They did not find that the receptors were overexpressed and suggest a limited direct role for these hormones in the development of the myelolipoma [18]. However this conclusion is debatable.

To our knowledge less than 20 cases of adrenal myelolipomas have been reported in patients with CAH to date [7–10, 16, 18–20]. Jaresch et al. found adrenal masses in 83% of CAH patients and 45% of heterozygous CYP21 mutation carriers, which is high compared to the general population [13]. However, their study included only 22 homozygous CAH patients and 20 heterozygous siblings. Conversely, series of adrenal incidentalomas have been tested for germline *CYP21* mutations, showing inconsistent results which could be due to small samples [12, 13, 15]. Consequently, it appears that adrenal masses are common in CAH, but that CAH is not a common cause of adrenal masses. 

The diagnosis of CAH in a patient with an incidental adrenal mass is important as the patient is at risk of adrenal crises [21]. Such patients may benefit from glucocorticoid therapy and should at least be equipped with emergency medication and a medical alert card. Patient 1 had a blunted cortisol response to ACTH stimulation, but he had managed well for 30 years without steroids, working full-time and did not want to take steroids because of the psychological side effects he experienced during adolescence. He was nonetheless advised to take steroids under stressful situations. Patient 2 had higher basal level of cortisol, but suboptimal cortisol response to ACTH stimulation. He had symptoms of cortisol deficiency, and his condition improved considerably after treatment with prednisolone was commenced. He had a low normal testosterone level and tenderness of the testicles. This could be the result of testicular adrenal rest tumors but was not investigated further. Whether glucocorticoid suppression therapy in adult age halts further growth of the tumor is uncertain. In our patients the tumors did not grow over four to nine year observation periods despite high ACTH levels. Conversely, Rajput et al. described progressive evolution of an adrenal myelolipoma in a woman with CAH who was treated with prednisolone, although taken irregularly, rendering the level of 17-OH-progesterone persistently elevated [19]. 

In conclusion, congenital adrenal hyperplasia, per se, should be included in the differential diagnosis of adrenal masses. If suspected clinically, assay of cortisol, ACTH, and 17OH-progesterone should be performed. Adrenal tumors are common in CAH and might require surgical removal, but myelolipomas are benign tumors that usually can be left untreated. Whether, treatment of CAH halts myelolipoma growth is uncertain.

## Figures and Tables

**Table 1:** Myelolipoma: baseline measurements and endocrinological test results.

	Patient 1	Patient 2	Normal
Baseline			

ACTH, pmol/L	6–32.8*	10.9–94.2	(2–13)
Cortisol, nmol/L	210	387	(250–750)
17 OH-progesterone, nmol/L	>605	445	(<6)
DHEAS, *μ*mol/L	14.4	1.8	(6–12)
Androstendione, nmol/L	43.8	10.9	(<5.6)
Testosterone, nmol/L	11.7	10.1	(10–40)

ACTH stimulation**			

Cortisol 30 minutes, nmol/L	211	409	
Cortisol 60 minutes, nmol/L	231	412	(>550)
17 OH-progesterone 30 minutes, nmol/L	>605	445	
17 OH-progesterone 60 minutes, nmol/L	>605	560	(<30)

Dexamethasone suppression***			

Cortisol, nmol/L	22	45	(<50)
17 OH-progesterone, nmol/L	14.3	18.8	

*Most values in the higher range. **Corticotropin (250 ug i.v.). ***1 mg overnight test in Patient 2 and 0.5 mg × 4 for 2 days in Patient 1.

## References

[1] Young WF (2007). The incidentally discovered adrenal mass. *The New England Journal of Medicine*.

[2] Kenney PJ, Wagner BJ, Rao P, Heffess CS (1998). Myelolipoma: CT and pathologic features. *Radiology*.

[3] Lezoche E, Guerrieri M, Crosta F (2008). Perioperative results of 214 laparoscopic adrenalectomies by anterior transperitoneal approach. *Surgical Endoscopy*.

[4] Mansmann G, Lau J, Balk E, Rothberg M, Miyachi Y, Bornstein SR (2004). The clinically inapparent adrenal mass: update in diagnosis and management. *Endocrine Reviews*.

[5] Rao P, Kenney PJ, Wagner BJ, Davidson AJ (1997). Imaging and pathologic features of myelolipoma. *Radiographics*.

[6] Hisamatsu H, Sakai H, Tsuda S, Shigematsu K, Kanetake H (2004). Combined adrenal adenoma and myelolipoma in a patient with Cushing's syndrome: case report and review of the literature. *International Journal of Urology*.

[7] Ravichandran R, Lafferty F, McGinniss MJ, Taylor HC (1996). Congenital adrenal hyperplasia presenting as massive adrenal incidentalomas in the sixth decade of life: report of two patients with 21-hydroxylase deficiency. *The Journal of Clinical Endocrinology & Metabolism*.

[8] Sakaki M, Izaki H, Fukumori T, Taue R, Kishimoto T, Kanayama H-O (2006). Bilateral adrenal myelolipoma associated with adrenogenital syndrome. *International Journal of Urology*.

[9] Umpierrez MB, Fackler S, Umpierrez GE, Rubin J (1997). Adrenal myelolipoma associated with endocrine dysfunction: review of the literature. *American Journal of the Medical Sciences*.

[10] Allison KH, Mann GN, Norwood TH, Rubin BP (2003). An unusual case of multiple giant myelolipomas: clinical and pathogenetic implications. *Endocrine Pathology*.

[11] Oliva A, Duarte B, Hammadeh R, Ghosh L, Baker RJ (1988). Myelolipoma and endocrine dysfunction. *Surgery*.

[12] Baumgartner-Parzer SM, Pauschenwein S, Waldhäusl W, Pölzler K, Nowotny P, Vierhapper H (2002). Increased prevalence of heterozygous 21-OH germline mutations in patients with adrenal incidentalomas. *Clinical Endocrinology*.

[13] Jaresch S, Kornely E, Kley H-K, Schlaghecke R (1992). Adrenal incidentaloma and patients with homozygous or heterozygous congenital adrenal hyperplasia. *The Journal of Clinical Endocrinology & Metabolism*.

[14] Kjellman M, Holst M, Backdahl M, Larsson C, Farnebo L-O, Wedell A (1999). No overrepresentation of congenital adrenal hyperplasia in patients with adrenocortical tumours. *Clinical Endocrinology*.

[15] Patocs A, Toth M, Barta C (2002). Hormonal evaluation and mutation screening for steroid 21-hydroxylase deficiency in patients with unilateral and bilateral adrenal incidentalomas. *European Journal of Endocrinology*.

[16] Pang S, Becker D, Cotelingam J (1981). Adrenocortical tumor in a patient with congenital adrenal hyperplasia due to 21-hydroxylase deficiency. *Pediatrics*.

[17] Selye H, Stone H (1950). Hormonally induced transformation of adrenal into myeloid tissue. *American Journal of Pathology*.

[18] Hagiwara H, Usui T, Kimura T (2008). Lack of ACTH and androgen receptor expression in a giant adrenal myelolipoma associated with 21-hydroxylase deficiency. *Endocrine Pathology*.

[19] Rajput RM, Bhansali AM, Khandelwal NM, Radotra BD (2007). Evolution of adrenal myelolipoma in a patient with congenital adrenal hyperplasia. *Endocrinologist*.

[20] Dell’Avanzato R, Gastaldi F, Giovanni C (2009). Giant symptomatic myelolipoma of the right adrenal gland: a case report. *Chirurgia Italiana*.

[21] Merke DP (2008). Approach to the adult with congenital adrenal hyperplasia due to 21-hydroxylase deficiency. *The Journal of Clinical Endocrinology & Metabolism*.

